# Differential influences of LDL cholesterol on functional outcomes after intravenous thrombolysis according to prestroke statin use

**DOI:** 10.1038/s41598-022-19852-8

**Published:** 2022-09-14

**Authors:** You-Ri Kang, Joon-Tae Kim, Ji Sung Lee, Beom Joon Kim, Kyusik Kang, Soo Joo Lee, Jae Guk Kim, Jae-Kwan Cha, Dae-Hyun Kim, Tai Hwan Park, Kyung Bok Lee, Jun Lee, Keun-Sik Hong, Yong-Jin Cho, Hong-Kyun Park, Byung-Chul Lee, Kyung-Ho Yu, Mi Sun Oh, Dong-Eog Kim, Wi-Sun Ryu, Jay Chol Choi, Jee-Hyun Kwon, Wook-Joo Kim, Dong-Ick Shin, Sung Il Sohn, Jeong-Ho Hong, Man-Seok Park, Kang-Ho Choi, Ki-Hyun Cho, Jong-Moo Park, Sang-Hwa Lee, Juneyoung Lee, Hee-Joon Bae

**Affiliations:** 1 Department of Neurology, Chonnam National University School of Medicine, Gwangju-Jeonnam Regional Cerebrovascular Center, Chonnam National University Hospital, 42 Jebongro, Dong-gu, Gwangju, 61469 Korea; 2 Clinical Research Center, Asan Institute for Life Sciences, Asan Medical Center, University of Ulsan College of Medicine, Seoul, Korea; 3 Department of Neurology, Cerebrovascular Center, Seoul National University Bundang Hospital, Seoul National University College of Medicine, 82, Gumi-ro 173 Beon-gil, Bundang-gu, Seongnam-si, 13620 Gyeonggi-do Korea; 4 Department of Neurology, Nowon Eulji Medical Center, Eulji University School of Medicine, Seoul, Korea; 5 Department of Neurology, Eulji University Hospital, Eulji University, Daejeon, Korea; 6 Department of Neurology, Dong-A University Hospital, Busan, Korea; 7 Department of Neurology, Seoul Medical Center, Seoul, Korea; 8 Department of Neurology, Soonchunhyang University Hospital, Seoul, Korea; 9 Department of Neurology, Yeungnam University Hospital, Daegu, Korea; 10 Department of Neurology, Ilsan Paik Hospital, Inje University, Goyang, Korea; 11 Department of Neurology, Hallym University Sacred Heart Hospital, Anyang, Korea; 12 Department of Neurology, Dongguk University Ilsan Hospital, Goyang, Korea; 13 Department of Neurology, Jeju National University Hospital, Jeju National University School of Medicine, Jeju, Korea; 14 Department of Neurology, Ulsan University College of Medicine, Ulsan, Korea; 15 Department of Neurology, Chungbuk National University Hospital, Cheongju, Korea; 16 Department of Neurology, Keimyung University Dongsan Medical Center, Daegu, Korea; 17 Department of Neurology, Uijeongbu Eulji Medical Center, Eulji University School of Medicine, Uijeongbu-si, Korea; 18 Department of Neurology, Department of Neurology, Hallym University Chuncheon Sacred Heart Hospital, Chuncheon-si, Gangwon-do Republic of Korea; 19 Department of Biostatistics, Korea University College of Medicine, Seoul, Korea

**Keywords:** Neurology, Neurological disorders, Stroke

## Abstract

This study aimed to elucidate whether low-density lipoprotein cholesterol (LDL-C) levels differentially affect functional outcomes after intravenous thrombolysis (IVT) depending on prestroke statin use. Patients with acute ischemic stroke treated with IVT were categorized into low, intermediate, and high LDL-C groups based on LDL-C levels at admission (< 100/100–130/ > 130 mg/dl, respectively). Multivariable logistic regression analyses were performed to explore the relationships between LDL-C and clinical outcomes (good outcomes at 3 months, modified Rankin Scale scores 0–2). The interaction between LDL-C levels and prestroke statin use regarding functional outcomes was investigated. Among the 4711 patients (age, 67 ± 12 years; males, 62.1%) who met the eligibility criteria, compared with the high LDL-C group, the low and intermediate LDL-C groups were not associated with good outcomes at 3 months according to the multivariable analysis. A potential interaction between the LDL-C group and prestroke statin use on good outcomes at 3 months was observed (*P*_interaction_ = 0.07). Among patients with prestroke statin use, low (aOR 1.84 [1.04–3.26]) and intermediate (aOR 2.31 [1.20–4.47]) LDL-C groups were independently associated with a greater likelihood of having a 3-month good outcome. Our study showed that LDL-C was not associated with a 3-month good outcome, but prestroke statin use could modify the influence of LDL-C levels on functional outcomes after IVT.

## Introduction

Intravenous thrombolysis (IVT) with alteplase is an important treatment for acute ischemic stroke (AIS); while it is likely to increase the chance of a good functional outcome, approximately half of patients treated with IVT do not exhibit a major change, leading to moderate to severe disability or death^1^. This treatment also increases the risk of intracerebral hemorrhage (ICH)^2^. Predictors of a poor functional outcome or symptomatic ICH (SICH) after IVT have been investigated since the introduction of intravenous tissue plasminogen activator (IV-tPA)^3^, and studies are still ongoing.

The role of the lipid profile in functional outcomes following IVT remains controversial, though low-density lipoprotein cholesterol (LDL-C)-lowering treatment with statins is well known to reduce the risk of vascular events in patients with ischemic stroke^4,5^. Some studies reported an association between a low LDL-C level and an increased risk of poor functional outcomes or SICH after IVT^6,7^, while others did not^8^. Additionally, regarding the effects of statins apart from the lipid profile, previous studies have shown inconsistent results, including improved outcomes, an increased risk of SICH, or no effects^9–12^. However, these studies were limited because of analyses in relatively small populations or the lack of information regarding the relationship between prestroke statin use and LDL-C levels.

Thus, considering the inconclusive evidence for the effects of LDL-C levels and prestroke statin treatment on outcomes after IVT, the influence of the interaction between these factors may require further exploration. This study aimed to elucidate whether LDL-C levels differentially affected the functional outcomes after IVT depending on prestroke statin use.

## Methods

### Subjects

This study was a retrospective analysis of a prospective, multicenter, nationwide registry, the Clinical Research Center for Stroke-Korea-National Institute of Health (CRCS-K-NIH) registry, which is a web-based database of consecutive patients with acute stroke or transient ischemic attack (TIA) admitted to 16 academic hospitals in South Korea. Detailed information about the CRCS-K-NIH registry has been previously reported^13,14^. We identified patients treated with IVT in the CRCS-K dataset of patients with AIS admitted between April 2008 and November 2018. The inclusion criteria were as follows: (1) patients with AIS or lesion-positive TIA and (2) patients with AIS treated with IV-tPA. Patients treated with endovascular reperfusion therapy, including mechanical thrombectomy, and those without cholesterol profiles at admission were excluded. A detailed patient selection flowchart is shown in Supplemental Figure I.

### Ethics statement

Clinical information was collected from the CRCS-K-NIH registry with approval from the local institutional review boards of all the participating centers. A waiver for informed consent was provided because of subject anonymity and minimal risk to the participants. All methods were performed in accordance with relevant guidelines and regulations. The data used in this study are available upon reasonable request following the submission of a legitimate academic research proposal to be assessed by the CRCS-K-NIH steering committee.

### Data collection

Demographic, clinical, imaging, and laboratory data were prospectively collected as previously reported^13,14^. The details are provided in the Supplemental Methods. Briefly, the following data were directly obtained from the registry database: demographic information, medical history, medication history, acute treatment, in-hospital treatment, stroke characteristics, and laboratory data, including LDL-C levels. Lipid profiles were obtained in fasting state, including LDL-C, non-high-density lipoprotein cholesterol (HDL-C), and triglyceride (TG) levels. The study subjects were divided into 3 groups according to LDL-C levels for comparison: low LDL-C (< 100 mg/dl), intermediate LDL-C (100–130 mg/dl), and high LDL-C (> 130 mg/dl) groups^15^. For additional analyses, non- HDL-C and TG levels were also categorized into 3 groups based on previous studies: low non-HDL-C (< 130 mg/dl), normal non-HDL-C (130–160 mg/dl), and high non-HDL-C groups (> 160 mg/dl); low TG (< 80 mg/dl), normal TG (80–130 mg/dl), and high TG groups (> 130 mg/dl)^15,16^.

### Outcomes

The primary outcome was a good functional outcome (functional independency) at 3 months, defined as a modified Rankin Scale (mRS) score of 0–2. The secondary outcomes were SICH, defined as worsening of neurological status (increase in National Institutes of Health Stroke Scale [NIHSS] scores of 4 or more) with the appearance of new parenchymal hemorrhage (type 2) on brain imaging that was sufficient to cause neurological deterioration; the Safe Implementation of Thrombolysis in Stroke-Monitoring Study (SITS-MOST) criteria^17^ and death within 3 months.

### Statistical analysis

The details of the statistical analysis are described in the Supplemental Methods. Briefly, a binary logistic regression analysis using linear mixed models to account for the center effect was performed to explore the relationships between LDL-C groups and dichotomized clinical outcomes. Two adjusted models were constructed (Supplemental Methods). ORs and 95% CIs were estimated. The modifying effect of previous statin use on the relationships between LDL-C levels and clinical outcomes was explored by introducing an interaction term of previous statin use and LDL-C groups into the models. E-values were calculated as a sensitivity analysis to assess the potential effects of unmeasured confounders on the analysis^18^. The E-value estimates the minimum magnitude of association that would be required between an unmeasured confounder and both the exposure and outcome, conditional on measured covariates, to overcome the statistically significant effect observed in a study where residual confounding is a potential problem^18^. In addition, a restricted cubic spline function with three knots defined at LDL-C levels of 100 mg/dl, 130 mg/dl, and 200 mg/dl was used in the logistic regression model to explore the shape of the relationship between LDL-C levels and a good outcome at 3 months in patients stratified according to prestroke statin use. The LDL-C cutoff values (and 3 knots) used in our study were predetermined based on recommendations in the guidelines for the prevention of vascular events^15^. Two-sided *p*-values < 0.05 were considered significant. For interaction testing reflecting the known insensitivity of interaction testing, evidence of heterogeneity was considered present with *p*-values ≤ 0.10. Statistical analyses were performed with R software using the “rms” package (version 3.6.0, R Foundation for Statistical Computing, Vienna, Austria) and SAS version 9.4 (SAS Institute Inc., Cary, NC, USA).

### Ethical approval

The current study was approved by an institutional review board at Chonnam National University Hospital.

### Consent to participate

A waiver for informed consent was provided because of study subject anonymity and minimal risk to the participants by an institutional review board at Chonnam National University Hospital.

## Results

### General characteristics

Among 70,004 patients with acute stroke, 4711 patients (mean age, 67 ± 12 years; males, 62.1%) were eligible for the study (Supplemental Figure I). The median initial NIHSS score was 7 (IQR 4–13). The mean LDL-C level at admission was 109.9 ± 36.3 mg/dl. The proportions of the LDL-C groups were as follows: low LDL-C group (< 100 mg/dl), 42.1%; intermediate LDL-C group (100–130 mg/dl), 31.6%; and high LDL-C group (> 130 mg/dl), 26.3%.

The general characteristics of the patients stratified according to LDL-C groups are shown in Table 1. Among the three groups, the low LDL-C group was most likely to have vascular risk factors, including a history of stroke, coronary artery disease (CAD), and atrial fibrillation (AF), and to be taking medications including statins at stroke onset, while the highest incidences of dyslipidemia and recent smoking were observed in the high LDL-C group. In terms of stroke etiology, cardioembolism (CE) was most frequent in the low LDL-C group, whereas the highest proportions of large artery atherosclerosis and small vessel occlusion were observed in the high LDL-C group.

**Table 1:** General characteristics of subjects.

	LDL-C < 100 mg/dl	LDL-C 100–130 mg/dl	LDL-C > 130 mg/dl	*P*	*P*1^‡^	*P*2^‡^
*N*	1985	1487	1239			
Age, yr (mean, SD)	69.0 ± 12.2	67.1 ± 12.5	65.2 ± 12.8	< 0.001	< 0.001	< 0.001
Male	1267 (63.8)	934 (62.8)	727 (58.7)	0.01	> 0.99	0.01
Prestroke mRS score > 1	259 (13.0)	139 (9.3)	126 (10.2)	0.001	0.001	0.03
Initial NIHSS score	8 (4–14)	7 (4–12)	7 (4–12)	0.001	0.001	0.01
OTT, min (median, IQR)	130 (88–183)	130 (88–185)	130 (89–182)	0.92	0.91	0.98
DTT, min (median, IQR)	37 (25–53)	36 (24–50)	36 (23–49)	0.04	0.23	0.04
BMI (mean, SD)	23.3 ± 3.5	23.8 ± 3.4	23.9 ± 3.5	< 0.001	< 0.001	< 0.001
**Risk factors, n (%)**						
HTN	1,365 (68.8)	934 (62.8)	748 (60.4)	< 0.001	< 0.001	< 0.001
DM	613 (30.9)	387 (26.0)	290 (23.4)	< 0.001	0.004	< 0.001
Dyslipidemia	551 (27.8)	288 (19.4)	449 (36.2)	< 0.001	< 0.001	< 0.001
Smoking	545 (27.5)	471 (31.7)	436 (35.2)	< 0.001	0.01	< 0.001
AF	776 (39.1)	483 (32.5)	276 (22.3)	< 0.001	< 0.001	< 0.001
High risk of CE	681 (34.3)	410 (27.6)	233 (18.8)	< 0.001	< 0.001	< 0.001
Coronary artery diseases	295 (14.9)	107 (7.2)	77 (6.2)	< 0.001	< 0.001	< 0.001
Prior stroke	436 (22.0)	188 (12.6)	119 (9.6)	< 0.001	< 0.001	< 0.001
Prior TIA	37 (1.9)	29 (2.0)	15 (1.2)	0.27	> 0.99	0.30
PAD	17 (0.9)	8 (0.5)	3 (0.2)	0.08	0.54	0.06
**Medication history, n (%)**						
Antiplatelet agent	768 (38.7)	345 (23.2)	192 (15.5)	< 0.001	< 0.001	< 0.001
Anticoagulant	100 (5.0)	38 (2.6)	15 (1.2)	< 0.001	< 0.001	< 0.001
Antihypertensive agent	1,150 (57.9)	690 (46.4)	498 (40.2)	< 0.001	< 0.001	< 0.001
Antidiabetic agent	483 (24.3)	252 (16.9)	184 (14.9)	< 0.001	< 0.001	< 0.001
Statin	559 (28.2)	148 (10.0)	68 (5.5)	< 0.001	< 0.001	< 0.001
**TOAST, n (%) **				< 0.001	< 0.001	< 0.001
LAA	489 (24.6)	417 (28.0)	443 (35.8)			
SVO	152 (7.7)	184 (12.4)	180 (14.5)			
CE	780 (39.3)	477 (32.1)	289 (23.3)			
UD/OE	564 (28.4)	409 (27.5)	327 (26.4)			
**Laboratory findings**						
White blood cell count, 10^3^/uL	8.30 ± 3.11	8.38 ± 2.91	8.77 ± 3.12	< 0.001	0.69	< 0.001
Hemoglobin, g/dL	13.5 ± 1.9	13.8 ± 1.8	14.1 ± 1.7	< 0.001	< 0.001	< 0.001
Creatinine, mg/dL	1.06 ± 0.86	0.99 ± 0.74	0.92 ± 0.49	< 0.001	0.01	< 0.001
Glucose, mg/dL	140.9 ± 53.5	139.8 ± 53.9	140.7 ± 55.9	0.81	0.77	0.99
SBP, mmHg	145.2 ± 25.7	149.5 ± 26.2	153.3 ± 29.3	< 0.001	< 0.001	< 0.001
**Acute lesions, n (%)**						
Multiple territory infarcts	314 (15.8)	225 (15.1)	151 (12.2)	0.02	> 0.99	0.01
**Large artery disease, n (%)**				< 0.001	0.29	< 0.001
No stenosis	797 (40.2)	633 (42.6)	533 (43.0)			
Mild stenosis < 50%	112 (5.6)	100 (6.7)	124 (10.0)			
Significant stenosis ≥ 50%	286 (14.4)	213 (14.3)	192 (15.5)			
occlusion	790 (39.8)	541 (36.4)	390 (31.5)			
**In-hospital treatment, n (%)**						
Antiplatelet agent	1302 (65.6)	1,018 (68.5)	871 (70.3)	0.02	0.15	0.01
Anticoagulant	346 (17.4)	211 (14.2)	115 (9.3)	< 0.001	0.02	< 0.001
Antihypertensive agent	798 (40.2)	575 (38.7)	470 (37.9)	0.4	0.72	0.40
Antidiabetic agent	382 (19.2)	265 (17.8)	209 (16.9)	0.22	0.57	0.18
Statin	1525 (76.8)	1269 (85.3)	1114 (89.9)	< 0.001	< 0.001	< 0.001

mRS, modified Rankin Scale; NIHSS, National Institutes of Health Stroke Scale; OTT, onset-to-treatment time; DTT, door-to-treatment time; BMI, body mass index; HTN, hypertension; DM, diabetes mellitus; AF, atrial fibrillation; CE, cardioembolism; TIA, transient ischemic attack; PAD, peripheral arterial disease; TOAST, Trial of Org 10172 in Acute Stroke Treatment; LAA, large artery atherosclerosis; SVO, small vessel occlusion; UD/OE, undetermined/other etiology; SBP, systolic blood pressure.

*P*-values were calculated using the chi-square test, ANOVA and Kruskal–Wallis test.

^‡^Adjusted *P*-values were calculated using Pearson’s chi-square test and Fisher’s exact test with the Bonferroni adjustment method, the Dwass, Steel, Critchlow-Fligner multiple comparison method or Dunnett’s multiple comparison method.

P1, LDL-C < 100 mg/dl vs LDL-C 100–130 mg/dl; P2, LDL-C < 100 mg/dl versus LDL-C > 130 mg/dl.

When patients were subclassified according to prestroke statin treatment, similar results to those described above were observed in patients without prestroke statin use; while the high LDL-C group was most likely to have dyslipidemia and a recent smoking history, the low LDL-C group was most likely to have history of CAD, prior stroke, and history of peripheral artery disease and had the highest proportion of CE among the three groups (Supplemental Table I).

### Outcomes of LDL-C groups

Of the 4,711 patients, 2,717 (57.7%) had a favorable functional outcome (mRS score of 0–2) at 3 months. Eighty-six patients (1.8%) developed SICH, and 411 patients (8.7%) died. The mean LDL-C level was significantly higher in patients with good outcomes than in those without good outcomes (111.0 ± 35.2 mg/dl vs. 108.3 ± 37.7 mg/dl, *p* = 0.01). Accordingly, when compared by group, the low LDL-C group had the lowest rate of 3-month good outcomes (low vs intermediate vs high; 55.0% vs. 60.2% vs. 59.0%, respectively; *p* = 0.005). The highest mortality rate was observed in the low LDL-C group (10.6% vs. 7.7% vs. 7.0%, respectively; *p* < 0.01), while the rate of SICH did not differ among the 3 groups (1.7% vs. 1.6% vs. 2.3%, respectively; *p* = 0.40) (Table 2).

**Table 2:** Rates of outcomes in all patients and subgroups stratified according to prestroke statin use.

	LDL-C < 100 mg/dl	LDL-C 100–130 mg/dl	LDL-C > 130 mg/dl	*P*	*P* for Trend
**All patients**	1985	1487	1239		
mRS 0–2	1091 (55.0)	895 (60.2)	731 (59.0)	0.01	0.01
mRS 0–1	788 (39.7)	632 (42.5)	513 (41.4)	0.24	0.26
Death	210 (10.6)	114 (7.7)	87 (7.0)	0.001	< 0.001
SICH	34 (1.7)	24 (1.6)	28 (2.3)	0.40	0.31
**Prestroke statin use**	559	148	68		
mRS 0–2	329 (58.9)	99 (66.9)	31 (45.6)	0.01	0.41
mRS 0–1	248 (44.4)	74 (50.0)	18 (26.5)	0.01	0.10
Death	56 (10.0)	7 (4.7)	7 (10.3)	0.13	0.36
SICH	10 (1.8)	2 (1.4)	3 (4.4)	0.23	0.31
**No prestroke statin use**	1426	1339	1171		
mRS 0–2	762 (53.4)	796 (59.4)	700 (59.8)	0.001	0.001
mRS 0–1	540 (37.9)	558 (35.0)	495 (42.3)	0.04	0.02
Death	154 (10.8)	107 (8.0)	80 (6.8)	0.001	< 0.001
SICH	24 (1.7)	22 (1.6)	25 (2.1)	0.60	0.41

*P*-values were calculated using the chi-square test and Fisher’s exact test.

*P*-values for the trend were calculated using the Cochran-Armitage trend test.

Based on the unadjusted analyses, the low LDL-C group was less likely to have a good functional outcome at 3 months than the high LDL-C group (OR 0.85 [0.73–0.98]). However, according to the multivariable analysis, the low and intermediate LDL-C groups were not associated with a good outcome at 3 months compared with the high LDL-C group (Table 3). Death was not associated with LDL-C groups. Although no significant associations were observed between LDL-C groups and SICH in the unadjusted analysis, compared with the high LDL-C group, the low LDL-C group was associated with reduced odds of SICH (OR 0.58 [0.34–0.99]) after adjustment for relevant variables.

**Table 3:** Multivariable logistic regression analysis.

	mRS 0–2 at 3 months	*P*	*P* int	Model 1	*P*^†^	*P*^†^ int	Model 2	*P*^†^	*P*^†^ int
	Crude OR (95% CI)								
**All patients**			0.01			0.07			0.08
LDL-C < 100 mg/dl	0.85 (0.73–0.98)	0.02		1.02 (0.86–1.22)	0.81		1.01 (0.84–1.21)	0.90	
LDL-C 100–130 mg/dl	1.05 (0.90–1.23)	0.53		1.12 (0.93–1.34)	0.23		1.13 (0.94–1.35)	0.20	
LDL-C > 130 mg/dl	Ref			Ref			Ref		
**Prestroke statin use**									
LDL-C < 100 mg/dl	1.71 (1.03–2.83)	0.04		1.84 (1.04–3.26)	0.04		1.77 (0.99–3.16)	0.054	
LDL-C 100–130 mg/dl	2.41 (1.34–4.34)	0.003		2.31 (1.20–4.47)	0.01		2.33 (1.19–4.57)	0.01	
LDL-C > 130 mg/dl	Ref			Ref			Ref		
**No prestroke statin use**									
LDL-C < 100 mg/dl	0.77 (0.66–0.90)	0.001		0.97 (0.81–1.17)	0.77		0.97 (0.80–1.17)	0.75	
LDL-C 100–130 mg/dl	0.99 (0.84–1.16)	0.87		1.06 (0.88–1.27)	0.56		1.07 (0.88–1.29)	0.50	
LDL-C > 130 mg/dl	Ref			Ref			Ref		

Variables adjusted for an mRS score of 0–2: Model 1: age, male sex, BMI, NIHSS score, HTN, DM, dyslipidemia, AF, history of stroke, history of CAD, TOAST, prestroke statin use, and LDL-C groups (interaction between prestroke statin use and LDL-C groups); Model 2: age, male sex, BMI, NIHSS score, HTN, DM, dyslipidemia, AF, history of stroke, history of CAD, TOAST, SBP, glucose level, creatinine level, previous antiplatelet agent use, previous antihypertensive agent use, previous antidiabetic agent use, previous statin use, LAD, and LDL-C groups (interaction between prestroke statin use and LDL-C group).

Variables adjusted for SICH and death: age, male sex, NIHSS score, HTN, DM, AF, history of stroke, TOAST, prestroke statin use and LDL-C groups (interaction).

^†^*P*-values were calculated with multiple logistic regression models using generalized linear mixed models to account for the center effect (using a random intercept model).

The E-value for unmeasured confounders was calculated for the adjusted OR of the study populations (Supplemental Table II). The adjusted OR point estimates of 1.01 and 1.13 for achieving a good functional outcome at 3 months associated with low and intermediate LDL-C groups corresponded to E-values of 1.11 and 1.32, respectively, and for the confidence interval value closest to the null, the E-values were 1 and 1, respectively. Adjusted odds ratios for the independent associations of selected covariates with good outcomes at 3 months are shown in Supplemental Table III.

### Interaction of LDL-C groups and prestroke statin use

**Figure 1:**
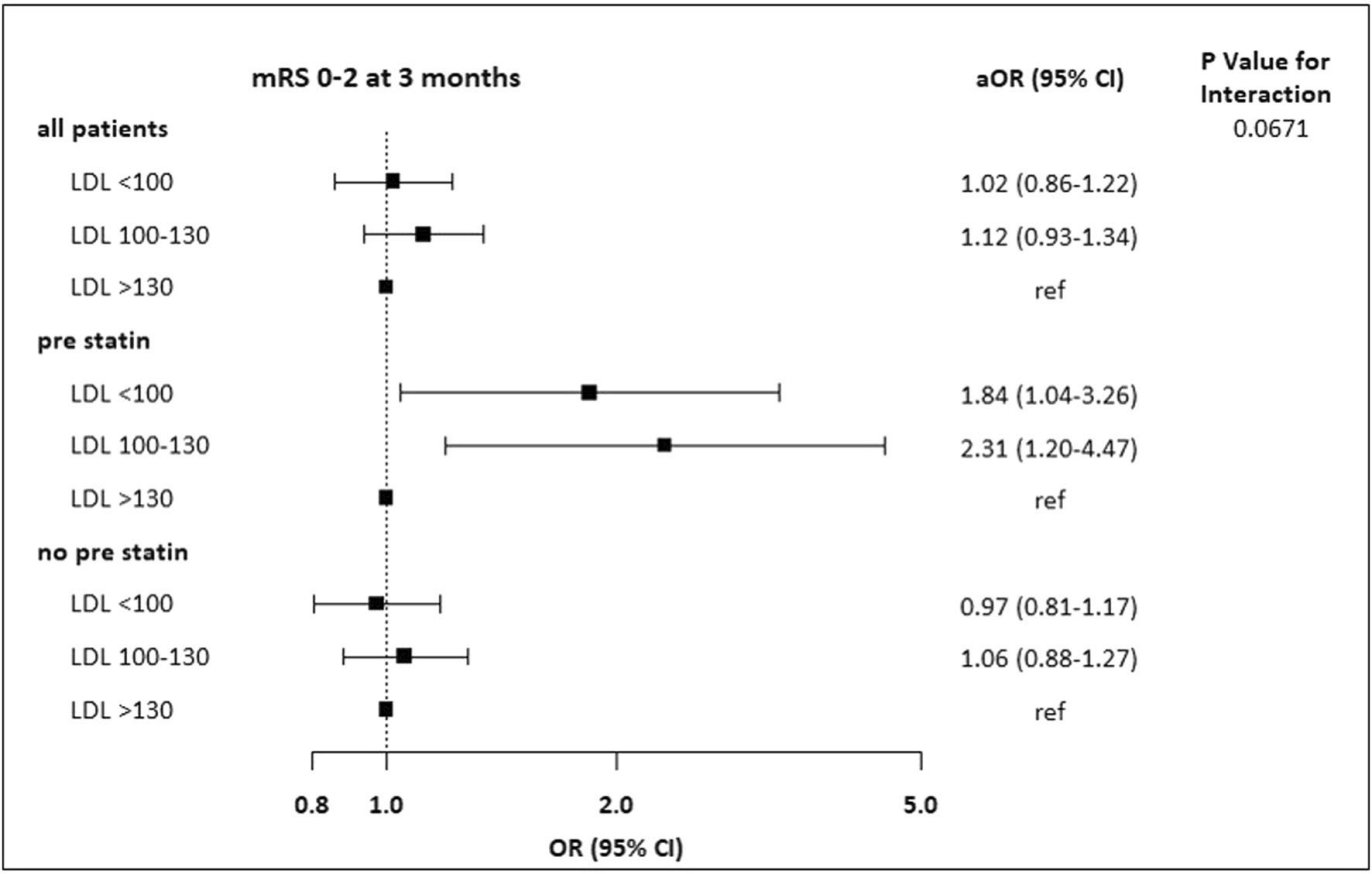
Associations of LDL-C groups with a good functional outcome at 3 months.

**Figure 2:**
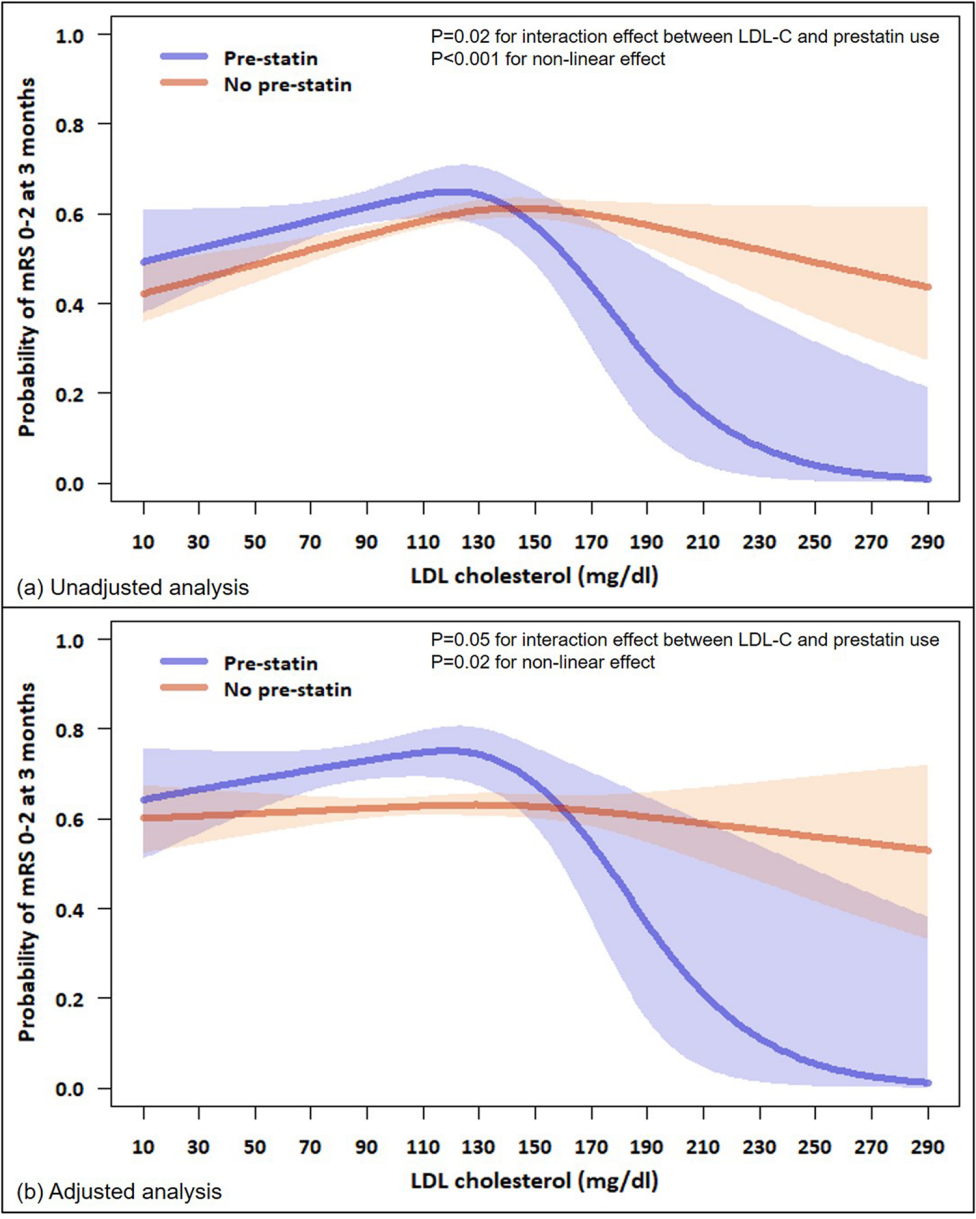
The unadjusted (**A**) and adjusted (**B**) predicted probabilities of the association between LDL-C levels and a good outcome at 3 months in patients stratified according to prestroke statin use.

The relationship between LDL-C groups and outcomes in patients stratified according to previous statin use is shown in Tables 2 and 3. Among patients with previous statin use, the lowest rate of good outcomes at 3 months was observed in the high LDL-C group (high vs. intermediate vs. low; 45.6% vs. 66.9% vs. 58.9%, respectively; *p* = 0.01), while among patients without previous statin use, the low LDL-C group was least likely to have a good outcome at 3 months (low vs. intermediate vs. high; 53.4% vs. 59.4% vs. 59.8%, respectively; *p* = 0.001). An effect of a potential interaction between the LDL-C groups and previous statin use on good outcomes at 3 months was observed (*P*_interaction_ = 0.07). Among patients with previous statin use, low (aOR 1.84 [1.04–3.26]) and intermediate (aOR 2.31 [1.20–4.47]) LDL-C groups were independently associated with a greater likelihood of having a good outcome at 3 months in the multivariable analysis compared with the high LDL-C group. However, among patients with no previous statin use, low or intermediate LDL-C groups were not significantly associated with a good outcome (Fig. 1). The shape of the relationship between LDL-C levels and good outcome was significantly different in patients stratified according to prestroke statin use (*P*_interaction_ = 0.02 and 0.05, unadjusted and adjusted analysis, respectively), and the association between LDL-C levels and a good outcome was non-linear (Fig. 2). In particular, among patients with prestroke statin use, the probability of a good outcome decreased substantially at LDL-C levels higher than 130 mg/dl. Regarding death and SICH, no interactions were observed between LDL-C groups and prestroke statin use (*P*_interaction_ = 0.40 and 0.75, respectively) (Supplemental Tables IV and V).

### Outcomes of other lipid profile groups

Regarding other lipid profiles (TG and non-HDL-C), the associations of outcomes of interest are shown in Supplemental Tables VI, VIII. After adjustment for covariates, the low TG group (aOR 0.83 [0.70–0.99]) was independently associated with a reduced chance of achieving a good outcome at 3 months (Supplemental Table VII), while the non-HDL-C groups were not associated with a good outcome at 3 months (Supplemental Table VIII).

## Discussion

In the analysis of over 4,500 patients with acute stroke treated with IV-tPA from a nationwide multicenter registry, the LDL-C level was not associated with functional outcomes at 3 months or death after IVT, consistent with previous studies showing no association between reduced LDL-C levels and functional outcomes^19,20^. However, a potential effect of the interaction between previous statin use and LDL-C groups was observed. In other words, the relationship between the LDL-C level and 3-month functional outcome varied depending on prior statin treatment. The lack of an association was consistently observed only for patients without prestroke statin treatment. Among the patients with prestroke statin use, the low and intermediate LDL-C groups were twice as likely to achieve a good functional outcome at 3 months compared with the high LDL-C group. These results suggest that modest lowering of LDL-C levels with prestroke statin treatment might help improve functional outcomes in patients with AIS after IVT.

Hypothetically, prestroke statin treatment is expected to exert neuroprotective effects by increasing angiogenesis, reducing clot formation or facilitating clot lysis, and upregulating endothelial nitric oxide synthase^21–23^. However, controversy exists regarding whether prestroke statin treatment improves the functional outcome after IVT in the clinic. As shown in Fig. 2, the predicted probability of a good outcome decreased sharply as the LDL-C level increased above a certain level in the prestroke statin user group. In contrast, this trend was not noticeable in the non-user group. The non-linear association of LDL-C levels with the functional outcome and the different effects of LDL-C levels on the functional outcomes of patients stratified according to prestroke statin treatment might partially explain the heterogeneity of the results of previous studies. Our results substantially improve our understanding of the clinical impact of LDL-C levels and statin treatment on patients with AIS treated with IVT.

Our results showed that the low LDL-C group (< 100 mg/dl) was most likely to have traditional vascular risk factors, a history of vascular diseases, and a medication use history including prestroke statin use. These findings are consistent with a previous study showing that prior statin use may be considered an indicator of demographic and vascular risk factors in IV-tPA-treated patients^11^. In our study, approximately 16% of all patients were on statin medication at the onset of stroke. Interestingly, 66.4% of prior statin users had LDL-C levels less than 100 mg/dl, in contrast to 36.2% of non-users. Accordingly, the low LDL-C group with prestroke statin treatment might be a well-controlled risk group, although the low LDL-C group appeared to have worse clinical risk profiles.

In contrast, it may be unexpected that the patients with no prestroke statin treatment in the low LDL-C group showed high vascular risk profiles. However, in the low LDL-C group, the no prestroke statin group, compared with that in prestroke statin group, had a less significant history of stroke (15.8% vs. 37.6%, respectively) and CAD (9.6% vs. 28.3%, respectively). The baseline characteristics of the patients with previous vascular diseases were mixed, which may have had an impact on stroke outcomes, thus potentially having an impact on the overall results of the research. More importantly, the low LDL-C group was more likely to have AF (40%) or CE subtype (39.7%) among the 3 LDL-C groups of patients with no prestroke statin use. Previously, low LDL-C levels were associated with a higher incidence of AF^24,25^. Therefore, among no prestroke statin users, history of stroke or history of CAD is likely to be related to AF pathology rather than LDL-C levels in the low LDL-C group. It is noteworthy to share real-world information regarding the characteristics of the LDL-C groups of AIS patients treated with IVT.

The results of our study showed that an LDL-C level of 100–130 mg/dl might have the highest predictive value for good outcome after IVT, especially in patients with prestroke statin use. Although the relationship of LDL-C levels with functional outcomes after IV-tPA treatment was not thoroughly investigated in previous studies, a U-shaped non-linear relationship between LDL-C levels and final infarct volume was observed in AIS patients treated with endovascular thrombectomy^26^. Also, the ‘lipid paradox’ might be partially related to our results. In the crude analysis, we observed that the low LDL-C group was more likely to have worse outcomes. The relationship between baseline LDL-C levels and early outcomes is complex, but LDL-C levels appear to be a potential indicator of the desired effect of statin pretreatment and might change the outcome after AIS. Further study would be warranted to confirm our results.

A long-standing dispute exists regarding the increased risk of SICH at lower LDL-C levels. Various reports have stated that low levels of cholesterol, including LDL-C, are associated with SICH or hemorrhagic transformation after IV or intra-arterial tPA treatment^6,19^, but others have reported no association^8,20^. In an analysis of 22,216 AIS patients treated with tPA^27^, statin treatment and LDL-C levels were not associated with the risk of SICH. In another study, a low level of LDL-C was independently associated with hemorrhagic transformation in patients with large artery atherosclerosis but not in patients with CE^28^. Unexpectedly, our study did not observe significant differences in the rates of SICH between LDL-C groups, but after adjustment for covariates, the low LDL-C group was 42% less likely to experience SICH than the high LDL-C group, regardless of the prestroke statin treatment. However, given the low incidence of SICH itself and the heterogeneous population, the results of the multivariable analysis should be interpreted with caution.

Our results provide additional information on the associations of other lipid profiles including non-HDL-C groups and TG groups with functional outcomes after IVT. While non-HDL-C groups were not associated with good outcomes at 3 months, the low TG group was less likely to have a good outcome at 3 months after IVT than the high TG group. The results for the non-HDL-C group were consistent with those of the LDL-C group, whereas the results of the TG group were not consistent. Previous studies have shown that a low TG concentration is associated with severe stroke and higher mortality after stroke^29,30^. However, further studies are warranted to explain these results.

Our study has several limitations. First, it is an inherently limited registry-based study of a patient cohort restricted to South Korea. Therefore, studies of other race-ethnicity groups are needed to confirm the generalizability of the results. Second, because of the retrospective design, we lacked clinical information on statin treatment profiles before starting statin therapy, the duration of therapy, the type and dosage of statins, and patient compliance. Moreover, the LDL-C cutoff values used in our study were based on recommendations in the guidelines for the prevention of vascular events, not the functional outcome. Third, despite multiple adjustments, unmeasured confounders might have influenced our findings. E-values were calculated as a sensitivity analysis to determine the likelihood that an unmeasured confounder existed that would negate the observed relationship between LDL-C levels and 3-month functional outcomes after IVT. This effect appears unlikely because the range of point estimates for the ORs for all known risk factors available in the data ranged from 0.65 to 2.33 (Supplemental Table III). The E-values for the confidence intervals closest to the null for the adjusted ORs for the associations between LDL-C groups (overall), prestroke statin users, and prestroke statin non-users ranged from 1 to 1.41. Fourth, we did not collect information about imaging predictors of functional outcomes such as the infarct volume, recanalization, collateral status, or leukoaraiosis severity. Finally, we cannot exclude the possibility that the acute phase reaction after stroke influenced lipid levels to some extent. Therefore, our results should be interpreted cautiously and confirmed in clinical trials.

## Conclusions

In the entire population of patients with AIS treated with IV-tPA in our study, the level of LDL-C was not associated with functional outcomes or death at 3 months. However, the LDL-C levels differentially affected functional outcomes in patients stratified according to prestroke statin treatment. In patients with prior statin treatment, lower LDL-C levels increased the likelihood of a good functional outcome, which was not consistently observed in patients without prior statin treatment. Based on these findings, the LDL-C level might be a potential predictor of functional outcomes after IVT in patients with AIS and a history of prestroke statin treatment.

## Supplementary Information

Supplementary Information.

## Data Availability

Corresponding author will provide the data, analytic methods, and study materials to other researchers upon reasonable request.
