# Supplementary material for: Differential influences of LDL cholesterol on functional outcomes after intravenous thrombolysis according to prestroke statin use
Source: Sci Rep. 2022 Sep 14;12:15478. doi: 10.1038/s41598-022-19852-8 (PMC9474509; doi:10.1038/s41598-022-19852-8)
Supplement: Supplementary file 1 — Supplementary Information. [file 41598_2022_19852_MOESM1_ESM.docx]

**ONLINE SUPPLEMENT**

**Title: Differential influences of LDL cholesterol on functional outcomes after intravenous thrombolysis according to prestroke statin use**

You-Ri Kang, MD,a Joon-Tae Kim, MD, PhD,a Ji Sung Lee, PhD,b Beom Joon Kim, MD, PhD,c Kyusik Kang, MD, PhD,d Soo Joo Lee, MD, PhD,e Jae Guk Kim, MD, e Jae-Kwan Cha, MD, PhD,f Dae-Hyun Kim, MD, PhD,f Tai Hwan Park, MD, PhD,g Kyung Bok Lee, MD, PhD,h Jun Lee, MD, PhD,i Keun-Sik Hong, MD, PhD,j Yong-Jin Cho, MD, PhD,j Hong-Kyun Park, MD,j Byung-Chul Lee, MD, PhD,k Kyung-Ho Yu, MD, PhD,k Mi Sun Oh, MD, PhD, k Dong-Eog Kim, MD, PhD,l Wi-Sun Ryu, MD,l Jay Chol Choi, MD, PhD,m Jee-Hyun Kwon, MD, PhD,n Wook-Joo Kim, MD, PhD,n Dong-Ick Shin, MD, PhD,o Sung Il Sohn, MD, PhD,p Jeong-Ho Hong, MD, PhD,p Man-Seok Park, MD, PhD,a Kang-Ho Choi, MD, PhD,a Ki-Hyun Cho, MD, PhD,a Jong-Moo Park, MD, PhD,q Sang-Hwa Lee, MD, PhD,r Juneyoung Lee, PhD,s Hee-Joon Bae, MD, PhD.c

aDepartment of Neurology, Chonnam National University Hospital, Chonnam National University Medical School, Gwangju, Korea

bClinical Research Center, Asan Institute for Life Sciences, Asan Medical Center, University of Ulsan College of Medicine, Seoul, Korea

cDepartment of Neurology, Cerebrovascular Center, Seoul National University Bundang Hospital, Seongnam, Korea

dDepartment of Neurology, Nowon Eulji Medical Center, Eulji University School of Medicine, Seoul, Korea

eDepartment of Neurology, Eulji University Hospital, Eulji University, Daejeon, Korea

fDepartment of Neurology, Dong-A University Hospital, Busan, Korea

gDepartment of Neurology, Seoul Medical Center, Seoul, Korea

hDepartment of Neurology, Soonchunhyang University Hospital, Seoul, Korea

iDepartment of Neurology, Yeungnam University Hospital, Daegu, Korea

jDepartment of Neurology, Ilsan Paik Hospital, Inje University, Goyang, Korea

kDepartment of Neurology, Hallym University Sacred Heart Hospital, Anyang, Korea

lDepartment of Neurology, Dongguk University Ilsan Hospital, Goyang, Korea

mDepartment of Neurology, Jeju National University Hospital, Jeju National University School of Medicine, Jeju, Korea

nDepartment of Neurology, Ulsan University College of Medicine, Ulsan, Korea

oDepartment of Neurology, Chungbuk National University Hospital, Cheongju, Korea

pDepartment of Neurology, Keimyung University Dongsan Medical Center, Daegu, Korea

qDepartment of Neurology, Uijeongbu Eulji Medical Center, Eulji University School of Medicine, Uijeongbu-si, Korea

rDepartment of Neurology, Department of Neurology, Hallym University Chuncheon Sacred Heart Hospital, Chuncheon-si, Gangwon-do, Republic of Korea

sDepartment of Biostatistics, Korea University College of Medicine, Seoul, Korea

Supplemental Materials:

Supplemental Methods

Supplemental Figures I

Supplemental Tables I-VIII

**SUPPLEMENTAL METHODS**

*Data collection*

Demographic, clinical, imaging, and laboratory data were prospectively collected. Baseline data, including National Institutes of Health Stroke Scale (NIHSS) scores, were collected from all patients, and the stroke subtypes were classified according to the Trial of Org 10172 in Acute Stroke Treatment (TOAST) criteria after complete diagnostic profiling. The following data were directly obtained from the registry database: (1) demographics, including age, sex, and body mass index; (2) medical history, including previous transient ischemic attack (TIA), previous stroke, previous coronary artery disease (CAD), previous peripheral artery disease (PAD), hypertension, diabetes mellitus, dyslipidemia, smoking (recent) habits, atrial fibrillation, congestive heart failure, and high potential cardioembolic sources; (3) medications, including previous antiplatelet medication, previous oral anticoagulant medication, previous antihypertensive medication, previous antidiabetic treatment, and previous statin medication; (4) stroke characteristics and acute treatment, including the time from onset to arrival, initial NIHSS scores, prestroke modified Rankin scale (mRS), ischemic stroke subtype according to the TOAST criteria, multiple territorial lesions on diffusion-weighted imaging (DWI), and large arterial disease, which was categorized as no stenosis, mild stenosis (<50%), significant stenosis ≥50%, and occlusion; (5) laboratory data, including white blood cell (WBC) counts, hemoglobin, creatinine serum levels, glucose level at presentation, low-density lipoprotein-cholesterol (LDL-C) level, non-high-density lipoprotein-cholesterol (non HDL-C) level, triglyceride (TG) level and systolic blood pressure; and (5) in-hospital treatment, including antiplatelet, anticoagulant, antihypertensive, statin, and antidiabetic therapies. For continuous variables, if fewer than 5% of the values were missing, the data were imputed as median values. The study subjects were divided into 3 groups according to the LDL-C level for comparison: low LDL-C (<100 mg/dl), normal LDL-C (100-130 mg/dl), and high LDL-C (>130 mg/dl) groups. For additional analyses, non HDL-C and TG levels were also categorized into 3 groups based on previous studies: low non-HDL-C (<130 mg/dl), normal non-HDL-C (130-160 mg/dl), and high non-HDL-C (>160 mg/dl) groups; low TG (<80 mg/dl), normal TG (80-130 mg/dl), and high TG (>130 mg/dl) groups.

*Workflow time metrics*

Time metrics were defined in the following 2 ways: 1) time from onset (time when the patient was last known to be well) to the start of IV-tPA treatment (OTT time), and 2) time from arrival to the start of IV-tPA treatment (DTT time).

*Outcome measures*

The primary outcome was a good functional outcome at 3 months (functional independency and a mRS score of 0–2). Other outcomes of interest were SICH, which was defined based on the Safe Implementation of Thrombolysis in Stroke-Monitoring Study (SITS-MOST) criteria; a decrease in the NIHSS score of 4 or more points, parenchymal hematoma type 2, and death.

*Statistical analysis*

The data are reported as percentages, means (standard deviations), or medians (IQRs), depending on the characteristics of the variable. Categorical variables were analyzed using Pearson’s chi-squared test or Fisher’s exact test, and continuous variables were analyzed using analysis of variance or the Kruskal-Wallis test, as appropriate. The following parameters had missing data that were substituted using median values: OTT (0.6%), DTT (0.6%), BMI (2.2%), creatinine (0.1%), hemoglobin (0.1%), WBC count (0.1%), and initial random glucose level (0.7%).

Baseline characteristics, workflow time metrics and outcomes were compared among the 3 LDL-C groups. A binary logistic regression analysis using linear mixed models to account for the center effect was performed to explore the relationships between LDL-C groups and dichotomized clinical outcomes. Two adjusted models were constructed. In the liberal model, adjustments were made for 2 predetermined variables with clinically relevant associations with outcome variables: age, male sex, baseline NIHSS score, HTN, DM, dyslipidemia, atrial fibrillation, history of stroke, history of CAD, TOAST classifications, prestroke statin use, and LDL-C groups (Model 1) and variables in Model 1 and BMI, SBP, glucose level, creatinine level, previous antiplatelet treatment, previous antihypertensive medication, previous antidiabetic treatment, and large artery disease (Model 2). In the conservative model, which was constructed because of fewer deaths and SICH, the model included only the candidate variables age, male sex, baseline NIHSS score, HTN, DM, atrial fibrillation, history of stroke, TOAST classification, prestroke statin use and LDL-C group. E-values were calculated as a sensitivity analysis to assess the potential effects of unmeasured confounders on the analysis. The E-value estimates the minimum magnitude of association that would be required between an unmeasured confounder and both the exposure and outcome, conditional on measured covariates, to overcome the statistically significant effect observed in a study where residual confounding is a potential problem.

In addition, a restricted cubic spline function with three knots defined as LDL-C levels of 100 mg/dl, 130 mg/dl, and 200 mg/dl in the logistic regression model was used to explore the shape of the relationship between LDL-C levels and a good outcome at 3 months in patients stratified according to prestroke statin use. The modifying effect of prestroke statin treatment on the relationships between LDL-C levels and clinical outcomes was explored by introducing an interaction term of prestroke statin use and LDL-C groups into the models. Two-sided p-values <0.05 were considered significant. For interaction testing, reflecting the known insensitivity of interaction testing, evidence of heterogeneity was considered present with p-values ≤0.10. Statistical analyses were performed with R software using the “rms” package (version 3.6.0, R Foundation for Statistical Computing, Vienna, Austria) and SAS version 9.4 (SAS Institute Inc., Cary, NC, USA).

Supplemental Figure I. Selection of the study population

70,004 patients with acute stroke in the CRCS-K-NIH registry (April 2008- November 2018)

62,210 patients excluded

Did not have ischemic stroke or lesion-positive TIA (n=6,517)

Not treated with IV-tPA therapy (n=55,693)

7,794 patients treated with IV-tPA

3,083 patients excluded

Combined treatment with EVT+IVT (n=2,255)

No lipid profile (n=274)

Lost to follow-up (no mRS information at 3 months) (n=554)

4,711 patients included in the analysis

Supplemental Table I. Characteristics of LDL-C groups stratified according to prestroke statin treatment

| Variables | No prestroke statin treatment | | | | Prestroke statin treatment | | | |
| --- | --- | --- | --- | --- | --- | --- | --- | --- |
|  | LDL-C <100 mg/dl | LDL-C 100-130 mg/dl | LDL-C >130 mg/dl | P | LDL-C <100 mg/dl | LDL-C 100-130 mg/dl | LDL-C >130 mg/dl | P |
| N | 1426 | 1339 | 1171 |  | 559 | 148 | 68 |  |
| age, yrs, mean (SD) | 68.5 (12.7) | 66.9 (12.7) | 65.1 (12.9) | <0.001 | 70.3 (10.6) | 68.9 (11.0) | 67.4 (10.9) | 0.06 |
| male, n (%) | 937 (65.7) | 850 (63.5) | 687 (58.7) | 0.001 | 330 (59.0) | 84 (56.8) | 40 (58.8) | 0.88 |
| prestroke mRS score >1 | 169 (11.9) | 125 (9.3) | 115 (9.8) | 0.07 | 90 (16.1) | 14 (9.5) | 11 (16.2) | 0.12 |
| initial NIHSS score, med (IQR) | 8 (4-14) | 7 (4-12) | 7 (4-12) | 0.0001 | 7 (4-14) | 7 (4-11) | 9 (5-15) | 0.06 |
| OTT, min, med (IQR) | 132 (90-184) | 132 (88-185) | 130 (89-182) | 0.86 | 125 (85-180) | 116 (86-180) | 125 (90-210) | 0.71 |
| DTT, min, med (IQR) | 36 (24-52) | 36 (24-50) | 36 (23-49) | 0.58 | 40 (28-54) | 36 (25-49) | 36 (24-53) | 0.11 |
| BMI, mean (SD) | 23.1 (3.5) | 23.7 (3.3) | 23.9 (3.5) | <0.001 | 23.8 (3.3) | 24.3 (3.5) | 24.2 (4.2) | 0.28 |
| risk factors |  |  |  |  |  |  |  |  |
| HTN | 898 (63.0) | 811 (60.6) | 696 (59.4) | 0.16 | 467 (83.5) | 123 (83.1) | 52 (76.5) | 0.34 |
| DM | 389 (27.3) | 329 (24.6) | 261 (22.3) | 0.01 | 224 (40.1) | 58 (39.2) | 29 (42.6) | 0.89 |
| dyslipidemia | 135 (9.5) | 178 (13.3) | 398 (34.0) | <0.001 | 416 (74.4) | 110 (74.3) | 51 (75.0) | 0.99 |
| smoking | 404 (28.3) | 421 (31.4) | 415 (35.4) | 0.001 | 141 (25.2) | 50 (33.8) | 21 (30.9) | 0.09 |
| AF | 570 (40.0) | 428 (32.0) | 259 (22.1) | <0.001 | 206 (36.9) | 55 (37.2) | 17 (25.0) | 0.15 |
| high risk of CE | 488 (34.2) | 362 (27.0) | 215 (18.4) | <0.001 | 193 (34.5) | 48 (32.4) | 18 (26.5) | 0.40 |
| coronary artery diseases | 137 (9.6) | 86 (6.4) | 63 (5.4) | <0.001 | 158 (28.3) | 21 (14.2) | 14 (20.6) | 0.001 |
| prior stroke | 226 (15.8) | 146 (10.9) | 99 (8.5) | <0.001 | 210 (37.6) | 42 (28.4) | 20 (29.4) | 0.07 |
| prior TIA | 22 (1.5) | 26 (1.9) | 12 (1.0) | 0.17 | 15 (2.7) | 3 (2.0) | 3 (4.4) | 0.61 |
| PAD | 13 (0.9) | 4 (0.3) | 2 (0.2) | 0.01 | 4 (0.7) | 4 (2.7) | 1 (1.5) | 0.10 |
| medication history |  |  |  |  |  |  |  |  |
| antiplatelet agent | 388 (27.2) | 257 (19.2) | 162 (13.8) | <0.001 | 380 (68.0) | 88 (59.5) | 30 (44.1) | 0.0002 |
| anticoagulant | 64 (4.5) | 30 (2.2) | 14 (1.2) | <0.001 | 36 (6.4) | 8 (5.4) | 1 (1.5) | 0.25 |
| Antihypertensive agent | 713 (50.0) | 578 (43.2) | 452 (38.6) | <0.001 | 437 (78.2) | 112 (75.7) | 46 (67.6) | 0.14 |
| antidiabetic agent | 302 (21.2) | 205 (15.3) | 162 (13.8) | <0.001 | 181 (32.4) | 47 (31.8) | 22 (32.4) | 0.99 |
| statin | - | - | - |  | 559 (100.0) | 148 (100.0) | 68 (100.0) |  |
| TOAST |  |  |  | <0.001 |  |  |  | 0.59 |
| LAA | 349 (24.5) | 383 (28.6) | 426 (36.4) |  | 140 (25.0) | 34 (23.0) | 17 (25.0) |  |
| SVO | 116 (8.1) | 169 (12.6) | 174 (14.9) |  | 36 (6.4) | 15 (10.1) | 6 (8.8) |  |
| CE | 566 (39.7) | 427 (31.9) | 268 (22.9) |  | 214 (38.3) | 50 (33.8) | 21 (30.9) |  |
| UD/OE | 395 (27.7) | 360 (26.9) | 303 (25.9) |  | 169 (30.2) | 49 (33.1) | 24 (35.3) |  |
| laboratory findings |  |  |  |  |  |  |  |  |
| white blood cell count | 8.37 (3.16) | 8.39 (2.94) | 8.75 (3.09) | 0.003 | 8.13 (2.98) | 8.32 (2.58) | 9.05 (3.62) | 0.05 |
| hemoglobin | 13.5 (2.0) | 13.8 (1.9) | 14.2 (1.7) | <0.001 | 13.4±1.8 | 13.7 (1.7) | 13.7 (2.0) | 0.11 |
| creatinine | 1.05 (0.81) | 0.99 (0.73) | 0.91 (0.49) | <0.001 | 1.10±0.97 | 1.05 (0.89) | 1.11 (0.50) | 0.83 |
| glucose | 138.5 (52.2) | 139.2 (53.0) | 139.4 (54.1) | 0.89 | 147.2±56.1 | 145.2 (61.5) | 163.2 (78.6) | 0.09 |
| SBP | 143.6 (25.8) | 149.6 (26.1) | 153.1 (29.2) | <0.001 | 149.3±24.8 | 149.0 (26.9) | 157.3 (31.3) | 0.05 |
| acute lesions |  |  |  |  |  |  |  |  |
| multiple territory infarcts | 225 (15.8) | 200 (14.9) | 144 (12.3) | 0.04 | 89 (15.9) | 25 (16.9) | 7 (10.3) | 0.43 |
| large artery disease |  |  |  | <0.001 |  |  |  | 0.06 |
| no stenosis | 568 (39.8) | 574 (42.9) | 502 (42.9) |  | 229 (41.0) | 59 (39.9) | 31 (45.6) |  |
| mild stenosis <50% | 86 (6.0) | 84 (6.3) | 118 (10.1) |  | 26 (4.7) | 16 (10.8) | 6 (8.8) |  |
| significant stenosis ≥50% | 196 (13.7) | 198 (14.8) | 184 (15.7) |  | 90 (16.1) | 15 (10.1) | 8 (11.8) |  |
| occlusion | 576 (40.4) | 483 (36.1) | 367 (31.3) |  | 214 (38.3) | 58 (39.2) | 23 (33.8) |  |
| in-hospital treatment |  |  |  |  |  |  |  |  |
| antiplatelet agent | 914 (64.1) | 913 (68.2) | 826 (70.5) | 0.002 | 388 (69.4) | 105 (70.9) | 45 (66.2) | 0.78 |
| anticoagulant | 248 (17.4) | 188 (14.0) | 110 (9.4) | <0.001 | 98 (17.5) | 23 (15.5) | 5 (7.4) | 0.10 |
| Antihypertensive agent | 529 (37.1) | 502 (37.5) | 438 (37.4) | 0.98 | 269 (48.1) | 73 (49.3) | 32 (47.1) | 0.95 |
| antidiabetic agent | 238 (16.7) | 229 (17.1) | 189 (16.1) | 0.81 | 144 (25.8) | 36 (24.3) | 20 (29.4) | 0.73 |
| statin | 1,041 (73.0) | 1,137 (84.9) | 1,053 (89.9) | <0.001 | 484 (86.6) | 132 (89.2) | 61 (89.7) | 0.58 |

Abbreviations: same as Table 1.

P-values were calculated using the chi-square test, Fisher’s exact test, ANOVA and the Kruskal-Wallis test.

Supplemental Table II. Multivariable logistic regression analysis

|  | Model 2 | P^†^ | P^†^ int | E-value  (Point Estimate)^a^ | E-value  (Confidence Interval)^b^ |
| --- | --- | --- | --- | --- | --- |
|  | OR (95% CI) |  |  |  |  |
| all patients |  |  | 0.08 |  |  |
| LDL-C <100 mg/dl | 1.01 (0.84-1.21) | 0.90 |  | 1.11 | 1 |
| LDL-C 100-130 mg/dl | 1.13 (0.94-1.35) | 0.20 |  | 1.32 | 1 |
| LDL-C >130 mg/dl | ref |  |  |  |  |
| prestroke statin use |  |  |  |  |  |
| LDL-C <100 mg/dl | 1.77 (0.99-3.16) | 0.05 |  | 1.99 | 1 |
| LDL-C 100-130 mg/dl | 2.33 (1.19-4.57) | 0.01 |  | 2.42 | 1.41 |
| LDL-C >130 mg/dl | ref |  |  |  |  |
| no prestroke statin use |  |  |  |  |  |
| LDL-C <100 mg/dl | 0.97 (0.80-1.17) | 0.75 |  | 1.14 | 1 |
| LDL-C 100-130 mg/dl | 1.07 (0.88-1.29) | 0.50 |  | 1.22 | 1 |
| LDL-C >130 mg/dl | ref |  |  |  |  |

The E-value represents the minimum magnitude of association required between an unmeasured confounder and both the exposure and outcome, conditional on measured covariates, to fully attenuate the observed exposure-outcome relationship.

The E-value was calculated using a publicly available online calculator. (Ref: Mathur MB, Ding P, Riddell CA, Vander Weele TJ. Web Site and R Package for Computing E-values. Epidemiology. 2018 Sep;29(5):e45-e47)

^a^ The point estimate E-value is the minimum odds ratio of an unmeasured confounder that would attenuate the point estimate of the association between LDL-C levels and an mRS score of 0-2 at 3 months to the null.

^b^ E-value for the confidence interval range closest to the null.

Supplemental Table III. Adjusted odds ratios for the independent association with a good outcome at 3 months of selected additional covariates included in Table 3, Model 2.

|  | OR (95% CI) | P-value |
| --- | --- | --- |
| Age (per 10-unit increase) | 0.65 (0.61-0.70) | <.01 |
| Male | 1.43 (1.24-1.65) | <.01 |
| BMI | 0.99 (0.97-1.01) | 0.45 |
| initial NIHSS score | 0.89 (0.88-0.91) | <.01 |
| risk factors |  |  |
| HTN | 0.82 (0.66-1.01) | 0.06 |
| DM | 0.94 (0.73-1.20) | 0.61 |
| dyslipidemia | 0.96 (0.80-1.15) | 0.66 |
| AF | 0.94 (0.75-1.19) | 0.60 |
| prior stroke | 0.65 (0.54-0.79) | <.01 |
| coronary artery diseases | 1.05 (0.83-1.32) | 0.69 |
| TOAST |  |  |
| LAA | 1 |  |
| SVO | 1.27 (0.96-1.68) | 0.09 |
| CE | 1.33 (1.01-1.75) | 0.04 |
| UD/OE | 1.16 (0.95-1.41) | 0.15 |
| SBP (per 10-unit increase) | 0.99 (0.96-1.01) | 0.29 |
| glucose (per 10-unit increase) | 0.97 (0.95-0.98) | <.01 |
| creatinine | 0.90 (0.83-0.99) | 0.03 |
| medication history |  |  |
| antiplatelet agent | 1.02 (0.85-1.21) | 0.86 |
| antihypertensive agent | 1.18 (0.96-1.45) | 0.12 |
| antidiabetic agent | 0.81 (0.62-1.06) | 0.12 |
| large artery disease |  |  |
| no stenosis |  |  |
| mild stenosis <50% | 0.89 (0.68-1.18) | 0.42 |
| significant stenosis >50% | 1.03 (0.82-1.29) | 0.82 |
| occlusion | 0.52 (0.44-0.62) | <.01 |
| LDL-C groups with prestroke statin use |  |  |
| LDL-C <100 mg/dl | 1.77 (0.99-3.16) | 0.05 |
| LDL-C 100-130 mg/dl | 2.33 (1.19-4.57) | 0.01 |
| LDL-C >130 mg/dl | 1 |  |
| LDL-C groups with no prestroke statin use |  |  |
| LDL-C <100 mg/dl | 0.97 (0.80-1.17) | 0.75 |
| LDL-C 100-130 mg/dl | 1.07 (0.88-1.29) | 0.50 |
| LDL-C >130 mg/dl | 1 |  |

Supplemental Table IV. Associations of LDL-C groups with death

|  | crude OR  (95% CI) | P | P int | adjusted OR  (95% CI) | P | P int |
| --- | --- | --- | --- | --- | --- | --- |
| all patients |  |  | 0.22 |  |  | 0.40 |
| LDL-C <100 mg/dl | 1.57 (1.21-2.03) | 0.001 |  | 1.12 (0.83-1.50) | 0.46 |  |
| LDL-C 100-130 mg/dl | 1.10 (0.82-1.47) | 0.52 |  | 0.91 (0.67-1.25) | 0.58 |  |
| LDL-C >130 mg/dl | ref |  |  | ref |  |  |
| prestroke statin use |  |  |  |  |  |  |
| LDL-C <100 mg/dl | 0.97 (0.42-2.22) | 0.94 |  | 1.00 (0.41-2.45) | 0.99 |  |
| LDL-C 100-130 mg/dl | 0.43 (0.15-1.29) | 0.13 |  | 0.47 (0.15-1.51) | 0.20 |  |
| LDL-C >130 mg/dl | ref |  |  | ref |  |  |
| no prestroke statin use |  |  |  |  |  |  |
| LDL-C <100 mg/dl | 1.65 (1.25-2.19) | 0.001 |  | 1.11 (0.82-1.52) | 0.50 |  |
| LDL-C 100-130 mg/dl | 1.18 (0.88-1.60) | 0.27 |  | 0.96 (0.69-1.33) | 0.81 |  |
| LDL-C >130 mg/dl | ref |  |  |  |  |  |

Adjusted variables: age, male sex, NIHSS score, HTN, DM, AF, history of stroke, TOAST, prestroke statin use and LDL-C groups (interaction).

^†^P-values were calculated using multivariable logistic regression analyses with generalized linear mixed models to account for the center effect (using a random intercept model).

Supplemental Table V. Associations of LDL-C groups with symptomatic ICH (SICH)

|  | crude OR  (95% CI) | P | P int | adjusted OR  (95% CI) | P | P int |
| --- | --- | --- | --- | --- | --- | --- |
| all patients |  |  | 0.55 |  |  | 0.75 |
| LDL-C <100 mg/dl | 0.75 (0.45-1.25) | 0.27 |  | 0.58 (0.34-0.99) | 0.04 |  |
| LDL-C 100-130 mg/dl | 0.71 (0.41-1.23) | 0.22 |  | 0.64 (0.36-1.11) | 0.11 |  |
| LDL-C >130 mg/dl | ref |  |  | ref |  |  |
| prestroke statin use |  |  |  |  |  |  |
| LDL-C <100 mg/dl | 0.39 (0.11-1.47) | 0.17 |  | 0.40 (0.11-1.52) | 0.18 |  |
| LDL-C 100-130 mg/dl | 0.30 (0.05-1.82) | 0.19 |  | 0.33 (0.05-2.06) | 0.24 |  |
| LDL-C >130 mg/dl | ref |  |  | ref |  |  |
| no prestroke statin use |  |  |  |  |  |  |
| LDL-C <100 mg/dl | 0.78 (0.45-1.38) | 0.40 |  | 0.60 (0.34-1.08) | 0.09 |  |
| LDL-C 100-130 mg/dl | 0.77 (0.43-1.37) | 0.37 |  | 0.68 (0.38-1.22) | 0.20 |  |
| LDL-C >130 mg/dl | ref |  |  | ref |  |  |

Adjusted variables: age, male sex, NIHSS score, HTN, DM, AF, history of stroke, TOAST, prestroke statin use and LDL-C groups (interaction).

^†^P-values were calculated using multivariable logistic regression analyses with generalized linear mixed models to account for the center effect (using a random intercept model).

Supplemental Table VI. Crude outcomes of other cholesterol profiles

|  | non-HDL-C <130 | 130-160 | >160 | P | P for Trend | TG <80 | 80-130 | >130 | P | P for Trend |
| --- | --- | --- | --- | --- | --- | --- | --- | --- | --- | --- |
| N | 2,498 | 1,240 | 973 |  |  | 1,566 | 1,718 | 1,427 |  |  |
| mRS 0-2 at 3months | 1,392 (55.7) | 733 (59.1) | 592 (60.8) | 0.01 | 0.003 | 808 (51.6) | 997 (58.0) | 912 (63.9) | <0.001 | <0.001 |
| death | 250 (10.0) | 93 (7.5) | 68 (7.0) | 0.004 | 0.002 | 171 (10.9) | 137 (8.0) | 103 (7.2) | 0.001 | 0.0003 |
| SICH | 43 (1.7) | 26 (2.1) | 17 (1.7) | 0.71 | 0.80 | 33 (2.1) | 30 (1.7) | 23 (1.6) | 0.57 | 0.31 |

P-values were calculated using the chi-square test and Fisher’s exact test.

P-values for the trend were calculated using the Cochran-Armitage trend test.

Supplemental Table VII. Association between outcomes and TG groups

|  | Crude OR (95% CI) | P | Model 1 | P | Model 2 | P |
| --- | --- | --- | --- | --- | --- | --- |
| mRS 0-2 at 3 months |  |  |  |  |  |  |
| TG <80 | 0.60 (0.52-0.70) | <0.001 | 0.83 (0.70-0.99) | 0.04 | 0.78 (0.66-0.94) | 0.01 |
| TG 80-130 | 0.78 (0.68-0.90) | 0.001 | 0.99 (0.84-1.17) | 0.91 | 0.94 (0.79-1.11) | 0.44 |
| TG >130 | ref |  | ref |  | ref |  |
| death |  |  |  |  |  |  |
| TG <80 | 1.58 (1.22-2.04) | 0.001 | 0.92 (0.69-1.23) | 0.57 |  |  |
| TG 80-130 | 1.11 (0.85-1.45) | 0.43 | 0.78 (0.58-1.05) | 0.10 |  |  |
| TG >130 | ref |  | ref |  |  |  |
| SICH |  |  |  |  |  |  |
| TG <80 | 1.31 (0.77-2.25) | 0.32 | 1.04 (0.59-1.82) | 0.90 |  |  |
| TG 80-130 | 1.08 (0.63-1.88) | 0.77 | 0.95 (0.54-1.65) | 0.85 |  |  |
| TG >130 | ref |  | ref |  |  |  |

Variables adjusted for an mRS score of 0-2; age, male sec, BMI, NIHSS score, HTN, DM, dyslipidemia, AF, history of stroke, history of CAD, TOAST, prestroke statin, and LDL-C levels; Model 2: age, male sex, BMI, NIHSS score, HTN, DM, dyslipidemia, AF, history of stroke, history of CAD, TOAST, SBP, glucose level, creatinine level, prior antiplatelet agent use, prior antihypertensive agent use, prior antidiabetic agent use, prestroke statin use, LASO, and LDL-C levels.

Variables adjusted for death/SICH; age, male sex, NIHSS score, HTN, DM, AF, history of stroke, TOAST, prestroke statin use and LDL-C groups (interaction).

P-values were calculated using multivariable logistic regression analyses with generalized linear mixed models to account for the center effect (using a random intercept model).

Supplemental Table VIII. Association between outcomes and non-HDL-C groups

|  | Crude OR (95% CI) | P | Model 1 | P | Model 2 | P |
| --- | --- | --- | --- | --- | --- | --- |
| mRS 0-2 at 3 months |  |  |  |  |  |  |
| non-HDL <130 | 0.81 (0.70-0.94) | 0.01 | 0.97 (0.80-1.16) | 0.73 | 0.94 (0.78-1.14) | 0.54 |
| non-HDL 130-160 | 0.93 (0.78-1.10) | 0.41 | 0.98 (0.80-1.19) | 0.83 | 0.97 (0.79-1.18) | 0.74 |
| non-HDL >160 | ref |  | ref |  | ref |  |
| death |  |  |  |  |  |  |
| non-HDL <130 | 1.48 (1.12-1.96) | 0.01 | 1.05 (0.77-1.44) | 0.74 |  |  |
| non-HDL 130-160 | 1.08 (0.78-1.49) | 0.65 | 0.94 (0.66-1.35) | 0.75 |  |  |
| non-HDL >160 | Ref |  | ref |  |  |  |
| SICH |  |  |  |  |  |  |
| non-HDL <130 | 0.98 (0.56-1.74) | 0.96 | 0.79 (0.44-1.42) | 0.42 |  |  |
| non-HDL 130-160 | 1.20 (0.65-2.23) | 0.55 | 1.12 (0.60-2.09) | 0.72 |  |  |
| non-HDL >160 | ref |  | ref |  |  |  |

Variables adjusted for an mRS score of 0-2; age, male sex, BMI, NIHSS score, HTN, DM, dyslipidemia, AF, history of stroke, history of CAD, TOAST, prestroke statin use, and LDL-C levels; Model 2: age, male sex, BMI, NIHSS score, HTN, DM, dyslipidemia, AF, history of stroke, history of CAD, TOAST, SBP, glucose level, creatinine level, prior antiplatelet agent use, prior antihypertensive agent use, prior antidiabetic agent use, prestroke statin use, LASO, and LDL-C levels.

Variables adjusted for death/SICH; age, male sex, NIHSS score, HTN, DM, AF, history of stroke, TOAST, prestroke statin use and LDL-C groups (interaction).

P-values were calculated using multivariable logistic regression analyses with generalized linear mixed models to account for the center effect (using a random intercept model).
